# Evaluating the implementation of rigid sterilization containers in a Dutch central sterile supply department: a scenario-based cost and workflow analysis

**DOI:** 10.1186/s12913-026-14391-8

**Published:** 2026-03-26

**Authors:** I. A. Kottink, A. C. Noort, J. W. Ongerboer de Visser, H. A. Cense

**Affiliations:** 1 Faculty of Economics and Business, University of Groningen, Groningen, The Netherlands; 2 Clinium BV, Amsterdam, The Netherlands; 3 Red Cross Hospital, Beverwijk, The Netherlands

**Keywords:** Central sterile supply department, Rigid sterilization containers, Workflow analysis, Healthcare sustainability, Cost analysis, Infrastructure planning

## Abstract

**Background:**

The transition from single-use blue wrap to rigid sterilization containers is becoming more common in healthcare settings to reduce packaging waste and align with sustainability goals. However, there is a lack of comprehensive evaluations that integrate workflow dynamics, capacity implications, and financial outcomes. This study investigates the operational and economic impacts of implementing rigid sterilization containers in a high-volume central sterile supply department.

**Methods:**

A comparative, scenario-based analysis was conducted using detailed workflow mapping and cost modeling. Operational data was collected over three weeks to establish baseline metrics for packaging time, autoclave loading efficiency, and washer-disinfector utilization. The financial analysis incorporated equipment investment, labor, consumable costs, and infrastructure needs under different implementation scenarios.

**Results:**

Introducing rigid sterilization containers reduced the average packaging time per instrument set by 61% and increased the autoclave capacity per cycle by 25%. The sterilization cost per set decreased by 40.4%, from €2.75 to €1.64. Based on typical usage patterns, the breakeven point for the initial investment was reached after approximately six years. However, the transition introduced new pressure points in the cleaning process, particularly when using large-capacity washer-disinfectors. Maintaining uninterrupted processing required either the addition of a new washer-disinfector or targeted workflow interventions.

**Conclusion:**

Rigid sterilization containers can deliver substantial efficiency improvements and cost savings, supporting their role as a viable, sustainable alternative to disposable wrapping materials in large-scale sterilization services. However, these benefits depend on system-wide coordination involving infrastructure adjustments and process redesign. Strategic planning and collaboration across hospital departments are essential for successful implementation. Future research should explore the effects on clinical operations, supply chain performance, and environmental impact.

**Supplementary Information:**

The online version contains supplementary material available at 10.1186/s12913-026-14391-8.

## Background

The healthcare system in the Netherlands is renowned for its accessibility, equity, and clinical outcomes, which are ranked among the best in the world [1, 2]. However, the healthcare sector is also a significant contributor to environmental pollution, accounting for 4.2% of the national waste generation footprint and 13.1% of the material extraction footprint [3]. The operating room is a critical contributor to this issue because it is one of the most resource-intensive areas in hospitals [4]. Over the last few decades, the increased reliance on disposable products has significantly amplified operating rooms’ waste production, making them major contributors to total hospital waste [5].

The Central Sterile Supply Department (CSSD) is a pivotal but often overlooked contributor to this environmental burden. The CSSD manages the cleaning, disinfection, sterilization, and distribution of clinical medical equipment to surgical departments. The CSSD plays a critical role in infection control and ensuring the quality of medical care through its sterilization practices [6]. Through its role in infection control, the CSSD plays a vital role in medical quality and safety [6, 7]. However, its heavy reliance on disposable blue wrap for packaging sterile goods introduces substantial waste and recurring costs. Estimates indicate that blue wrap accounts for approximately 11.5% of total operating room (OR) waste [8, 9]. Therefore, addressing the environmental footprint of CSSD processes is essential to broader efforts toward sustainable, high-quality care.

Recent studies have begun to explore the financial and environmental benefits of replacing single-use blue wrap with rigid sterilization containers (RSCs) in CSSDs. Krohn et al. (2019) reported initial cost savings following such a transition in a German hospital but emphasized that initial investments can pose a substantial barrier, particularly for institutions with limited access to capital or infrastructure financing [10]. Similarly, Rizan et al. (2022) and Friedericy et al. (2021) quantified the carbon footprint of sterilization packaging methods, demonstrating that RSCs can significantly reduce emissions and long-term costs [11, 12]. These studies highlight the potential of reusable systems to align healthcare delivery with sustainability goals.

However, these existing studies have focused on per-unit environmental savings or retrospective cost comparisons without systematically addressing the operational preconditions for successful implementation. They rarely include dynamic capacity analysis, workflow redesign, or consideration of context-specific constraints, such as throughput variability or investment timelines. This study addresses this gap by providing a scenario-based evaluation of RSC adoption in a high-volume Dutch CSSD. Drawing on Lean Thinking principles and value stream mapping, the study focuses on three objectives: mapping current and future sterilization processes, identifying operational inefficiencies, and evaluating the impact of RSC implementation on workflow and costs.

In doing so, the study contributes to the growing literature on sustainable healthcare logistics and to ongoing policy and ethical debates on integrating environmental goals with operational and financial feasibility in hospital settings.

## Methods

### Study design

This study used a scenario-based workflow and cost analysis to evaluate the operational and financial impacts of implementing RSCs in a Dutch CSSD. The analysis compared a current-state scenario in which single-use blue wrap is used to sterilize surgical instruments with a future-state scenario in which RSCs are used. The goal was to assess the effects of this transition on throughput, equipment utilization, and costs.

### Setting

The study was conducted in 2024 at two CSSDs in the Netherlands. The primary study site was a privately operated CSSD that serves several hospitals. It was selected because of its detailed process documentation and expressed interest in sustainable innovations. This site was used to map and analyze the current scenario based on the use of single-use blue wrap.

The second CSSD, which had previously implemented RSCs, served as a reference case to inform the future-state scenario. Data collection at both sites included direct observations, semi-structured staff interviews, and extraction of key operational metrics. A summary of the topics discussed during these consultations is provided in Appendix A. This dual-site approach enabled realistic modeling of the transition from blue wrap to RSCs and supported comparative assessments of workflows, capacities, and costs.

### Data collection

Data collection focused on capturing operational and cost-related parameters relevant to the sterilization process, including instrument set turnover, average packaging times, and the loading capacity of washer-disinfectors and autoclaves.

At the primary CSSD, operational data were collected through direct observations, operational logs, and structured process measurements during three randomly selected, non-consecutive weeks in 2024. This sampling strategy was chosen to capture routine process characteristics while reducing the influence of short-term anomalies or incidental workload fluctuations. The observation period was used for detailed process mapping, time-motion measurements, and capacity assessment within a highly standardized CSSD environment.

Data for the future-state scenario involving reusable rigid sterilization containers (RSCs) were obtained from a second CSSD that had already implemented container-based workflows. Data collection at this site included on-site observations, semi-structured interviews with experienced staff, and review of technical specifications provided by container manufacturers. Packaging time was estimated based on semi-structured interviews with experienced CSSD staff at a reference site where container-based workflows were fully implemented. Staff consistently indicated that packaging with RSCs involves placing the instrument set into the container and closing the lid, without additional wrapping steps. Based on this input, average packaging time was estimated at 30 s per instrument set. Because the RSC packaging procedure is highly standardized, requires limited manual actions, and is largely independent of instrument set size or composition, packaging time was modelled as a deterministic parameter in the scenario-based analyses.

Instrument demand within the CSSD is largely derived from scheduled operating room (OR) activity, which in many hospitals is governed by a Master Surgical Schedule (MSS) or cyclic block scheduling system. These schedules allocate OR time to clinical specialties in fixed, repeating patterns over multi-week cycles, typically ranging from one to four weeks, to promote predictability and operational stability. The MSS is designed as a long-term, year-round planning framework, with only limited week-to-week variability introduced to improve system performance [13]. Consistent with findings from the operating room planning literature, demand under block scheduling systems is generally stable over time, with variability occurring primarily at the intra-day or case-mix level rather than across seasons [14, 15].

Given this structural stability in OR scheduling, the selected observation period was considered sufficient to characterize typical CSSD workflow patterns and associated process variability.

### Cost analysis

A cost comparison was conducted to assess the total cost per sterilization cycle for single-use blue wrap versus reusable sterilization containers (RSCs). The analysis included fixed costs, such as container acquisition, depreciation, and infrastructure adjustments, as well as variable costs, including materials, labor, water, and energy consumption. Scan log data were used to analyze packaging times for blue wrap. For the RSC scenario, average packaging time was derived from staff interviews and observations at the reference CSSD. Reductions in labor time were modelled as capacity-related efficiency gains within a standardized workflow, rather than as direct changes in staff remuneration.

To determine financial feasibility, a breakeven analysis was performed based on projected annual sterilization demand. This analysis examined how high-throughput volume spreads fixed investment costs. It should be noted that this breakeven analysis focused exclusively on direct, cycle-based cost differences and did not account for potential indirect savings, such as reductions in waste processing costs, lower repackaging error rates, or ergonomic improvements due to standardized container handling. Additionally, the analysis was based on a standardized implementation scenario in which a single type of RSC was used for all instrument sets included. Under this assumption, any instrument set can be processed in any available container, allowing for operational flexibility and avoiding the need to allocate dedicated containers to specific sets. Consequently, the total number of containers required was calculated based on the aggregate volume of sets and the average reuse rate rather than on a fixed one-to-one mapping between sets and containers.

Additionally, a sensitivity analysis was conducted to evaluate the robustness of the cost advantage of RSCs under varying assumptions. These assumptions included fluctuations in turnover volume, different container lifespans, and purchasing an additional large washer-disinfector. This approach provided a more nuanced understanding of the conditions under which RSCs could be a viable, economical alternative to blue wrap.

### Lean waste analysis

To evaluate the operational implications of transitioning from single-use blue wrap to RSCs, this study applied Lean Thinking principles, which were originally developed in the Toyota Production System [16]. Lean methodology focuses on understanding and improving process performance by identifying and analyzing three interrelated forms of waste: non-value-adding activities (muda), process variability (mura), and overburden of people or equipment (muri) [17]. This framework was used to systematically assess how changes in the packaging system affect workflow structure, resource use, and capacity within the central sterile services department (CSSD).

Value stream mapping (VSM) was employed as the primary methodological tool to document and analyze the sterilization workflow [18, 19]. VSM provides a structured, visual representation of process steps, material flows, information flows, and associated processing and waiting times. In healthcare settings, VSM is commonly used to identify delays, redundancies, and imbalances in workload distribution. In this study, VSM was applied to construct detailed current-state and future-state maps of internal CSSD operations. The scope of the analysis was limited to in-department processes and excluded downstream activities such as hospital transport logistics and intraoperative instrument handling.

In its descriptive role, VSM was used to quantify workflow characteristics in the current state, including task durations, queue times, batch sizes, and equipment utilization across decontamination, packaging, and sterilization stages. Inefficiencies in the existing workflow were identified through a combination of empirical time measurements, review of operational logs, and structured on-site observations. Each observed inefficiency was categorized according to the three Lean waste domains (muda, mura, and muri), providing a standardized framework for comparing process characteristics between scenarios.

In addition to its descriptive function, VSM served an analytical role by enabling a structured comparison between the blue wrap and RSC workflows. By mapping both current- and future-state processes, the analysis identified process steps that differed structurally between the two packaging systems and that directly affected labor input, equipment loading patterns, and process flow. These identified differences informed the selection and operationalization of workflow parameters used in subsequent scenario-based analyses of cost and capacity.

The future state scenario was developed using data from a CSSD that had transitioned to RSCs. Observations at this site were supplemented by interviews with experienced staff and technical specifications provided by container manufacturers. This combination of observational, qualitative, and technical input was used to model the expected workflow characteristics of RSC use in a comparable operational context, while maintaining consistency with the process scope defined for the current-state analysis. Grounding the future-state scenario in realistic assumptions and site-specific knowledge ensured that the modeled workflows remained actionable and relevant. This forward-looking value stream map enabled a structured comparison between current and projected operations, making it possible to assess the potential for reducing waste through RSC implementation.

Based on the VSM comparison, four key workflow parameters were selected to assess performance and capacity differences between the two scenarios: daily instrument set turnover, average packaging time per set, autoclave loading capacity, and washer-disinfector throughput. These parameters capture core aspects of operational load and resource utilization within the CSSD. Table 1 summarizes the data sources and estimation methods used for each parameter in the current and future states.

**Table 1:** Workflow parameters

Workflow parameter	Current state	Future state
Daily instrument set turnover	Determined using operational data logs over a three-week period at the primary CSSD.	Assumed to be equal to the current state, based on the expectation that clinical demand remains constant regardless of packaging method.
Average packaging time	Measured empirically through direct time observations during standard routines at the primary CSSD.	Estimated based on observations at the second CSSD and validated through interviews with experienced staff.
Autoclave loading capacity	Calculated through practical test loads and spatial assessment.	Estimated using container dimensions and autoclave specifications.
Washer-disinfector throughput	Derived from operational data: daily logs and observations, covering disinfection of transport baskets and trolleys.	Includes additional disinfection cycles for containers. Estimated based on container capacity and observations at the second CSSD.

The workflow parameters derived from the value stream maps formed the quantitative basis for subsequent scenario-based analyses. In this way, VSM functioned as both a structured method for documenting existing workflows and an analytical framework for isolating process elements affected by the transition from blue wrap to reusable sterilization containers.

## Results

### Workflow mapping and process structure

**Fig. 1:**
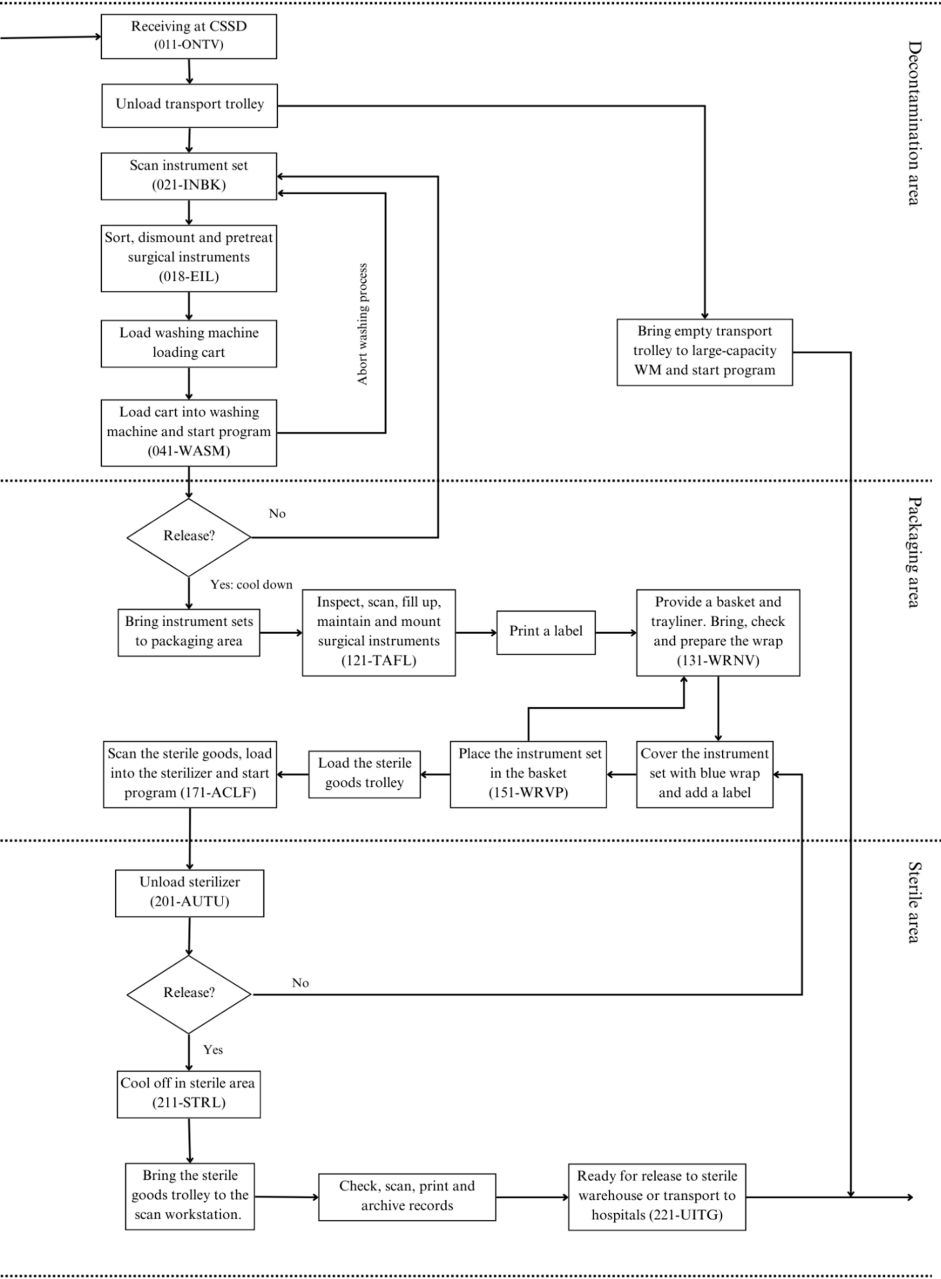
Current-state CSSD workflow using single-use blue wrap

**Fig. 2:**
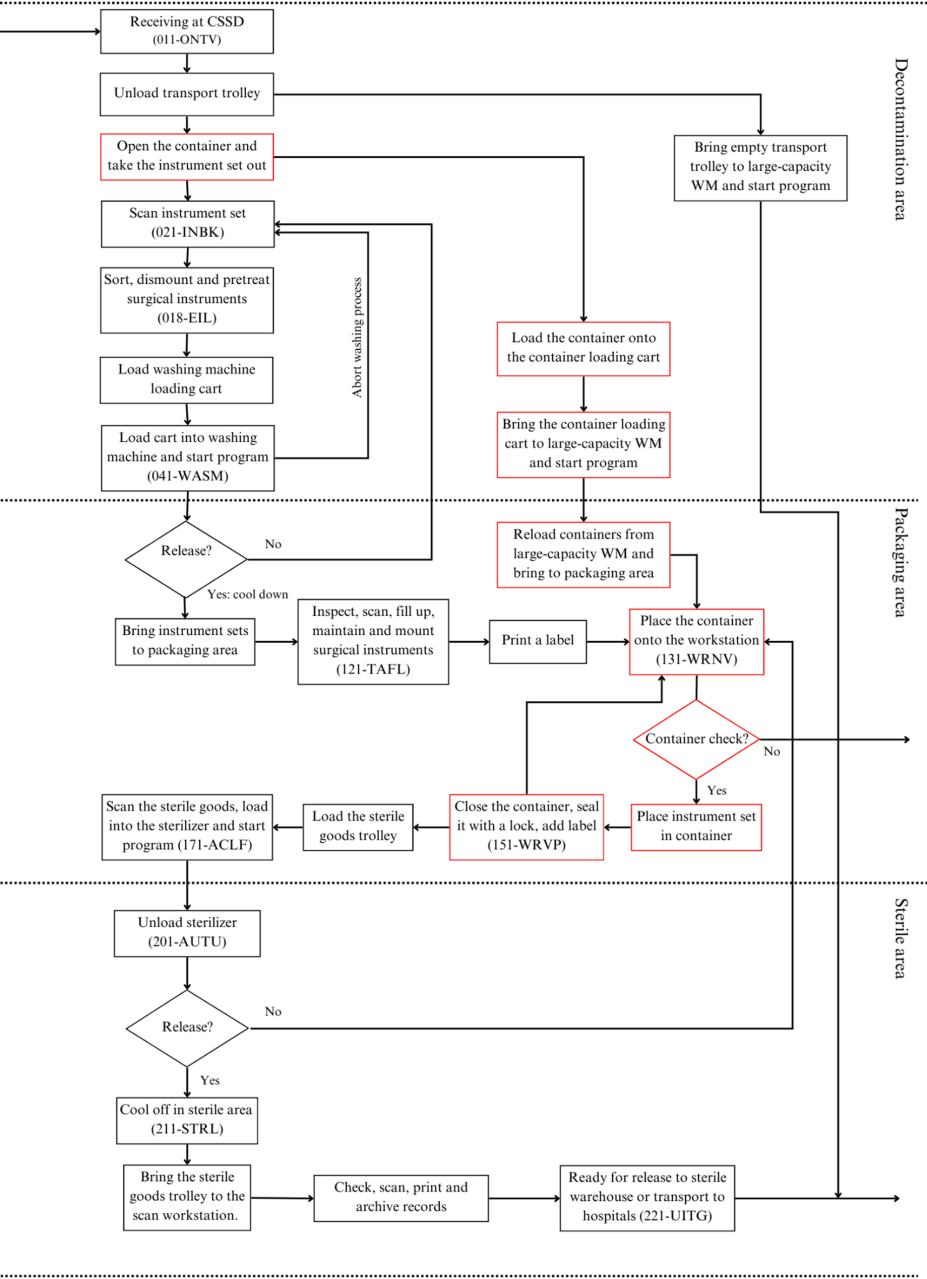
Future state CSSD workflow using RSCs: additional steps compared to the current state are shown in red

The current-state and future-state sterilization workflows were analyzed. The current process, which uses blue wrap, consists of 20 distinct sub-processes (Fig. 1). The future state scenario, which includes RSCs, has 24 sub-processes (Fig. 2). The additional steps, shown in red, mainly relate to container preparation, disinfection, and transport handling. These modifications reflect shifts in workflow complexity and resource requirements, providing a basis for evaluating logistical feasibility and efficiency.

#### Daily turnover and autoclave capacity

During a three-week period, an average of 552 instrument sets were processed per day. This corresponds to an estimated annual volume of 201,725 sets. Currently, all sets are packaged using single-use blue wrap with an average autoclave load of 10 sets per cycle, while the maximum loading capacity was 16 sets per cycle. Assuming full-capacity utilization, this results in approximately 12,116 autoclave cycles per year.

For the future state scenario, the autoclave capacity was recalculated based on the dimensions of the RSCs intended for use in the CSSD. Each container measures 592 × 291 × 115 mm. A spatial assessment of the autoclave chamber indicated that up to 20 full-size containers could be loaded per cycle (Table 2). This represents a 25% increase in per-cycle autoclave capacity relative to the current configuration. This increase is due to the containers’ uniform size and stackability, which allow for more efficient space utilization during sterilization.

**Table 2:** Autoclave utilization and capacity

	Blue wrap	RSCs
Daily turnover of instrument sets	552	552
Maximum autoclave utilization (sets/cycle)	16	20
Average autoclave utilization (sets/cycle)	10	N/A
Minimal daily autoclave cycles (if maximum loaded)	35	28

#### Average packaging time

In the current state using blue wrap, packaging time was derived from 750 scan log observations collected at the primary CSSD. These data demonstrated variability in packaging duration, reflecting differences in instrument set size and wrapping complexity. The mean packaging time was 77.6 s per set (SD = 28.3).

In the RSC scenario, packaging time was estimated at 30 s per instrument set, based on observations and semi-structured interviews with experienced staff at a reference CSSD using container-based workflows. Given the standardized nature of the container packaging procedure, this value was treated as deterministic value in the scenario-based analysis.

Compared to the current state, the estimated packaging time in the RSC scenario represents a 61% reduction in average packaging time per instrument set. These packaging time estimates were subsequently used to calculate the labor cost component in the cost comparison and breakeven analyses.

#### Washer-disinfector capacity

In the current CSSD configuration, six standard washer-disinfectors are used to clean surgical instrument sets. This will remain unaffected by the transition to RSCs. Additionally, the department operates two large-capacity washer-disinfectors (LCWDs) that are currently used to clean transport carts and baskets. Each LCWD can handle two carts or 24 baskets per cycle.

Under the blue wrap scenario, an average day (552 instrument sets) requires 11 cycles for carts and five cycles for baskets, for a total of 16 cycles. On high-volume days (860 sets), this increases to 18 cycles for carts and eight cycles for baskets, for a total of 26 cycles.

With the implementation of RSCs, transport baskets are no longer needed because containers allow for direct stacking and transport. However, each container must be cleaned after use, and an LCWD can handle 48 containers. Consequently, on an average day, the RSC scenario requires 11 cycles for carts and 12 cycles for containers, for a total of 23 LCWD cycles. On peak days, this increases to 18 cycles for carts and 18 cycles for containers, for a total of 36 cycles.

Table 3 summarizes the required LCWD cycles per day under both scenarios and illustrates the operational impact of adding container cleaning to the disinfection workflow.

**Table 3:** Estimated daily cycles in large-capacity washer-disinfector

	Blue wrap	RSCs
*Average day: 552 instrument sets*		
Transport carts (2 per cycle)	11	11
Transport baskets (24 per cycle)	5	-
Containers (48 per cycle)	-	12
**Total daily cycles**	**16**	**23**
*High volume day: 860 instrument sets*		
Transport carts (2 per cycle)	18	18
Transport baskets (24 per cycle)	8	-
Containers (48 per cycle)	-	18
**Total daily cycles**	**26**	**36**

### Cost analysis

The personnel cost calculation for each packaging option was based on packaging times and the CSSD personnel minute rate, which were 77.6 s for blue wrap and 30 s for RSCs, respectively. Consequently, the average personnel cost to package one instrument set was calculated to be €0.99 for blue wrap and €0.39 for RSCs. Material costs for the blue wrap were calculated at €1.41, including the cost of the two-layer wrap (€1.21), trayliner (€0.11), labels (€0.05), and tape with an indicator (€0.04). The material costs for RSCs were €0.89, including the cost of the seal (€0.17), labels and the depreciation cost of €0.67 per use. There was also an additional cost of €0.01 for repairs. Furthermore, about €0.22 per instrument set is attributable to water consumption costs, and about €0.13 is attributable to power consumption costs. RSCs had the lowest costs at €1.64 versus €2.75 for blue wrap, about 67.7% higher (see Table 4).

**Table 4:** Cost per instrument set, per cycle

Category		Blue wrap	RSCs
Personnel cost in €	CSSD	0.99	0.39
Material cost in €	Wrap (2 layers)	1.21	-
	Trayliner	0.11	-
	Labels	0.05	0.05
	Container seal	-	0.17
	Tape with indicator	0.04	-
	Container cost	-	0.67
Fixed costs in €	Water consumption	0.13	0.13
	Power consumption	0.22	0.22
	Container repair	-	0.01
Total cost excluding container cost			*0.97*
**Total cost in €**		**2.75**	**1.64**

A breakeven analysis was conducted to assess the financial implications of transitioning from single-use blue wrap to reusable storage containers (RSCs), using observed and modeled cost differences per sterilization cycle. The CSSD processes approximately 201,725 surgical instrument sets annually. Based on operational data, each container is used an average of 71 times per year. This means that 2,842 RSCs would be required for full implementation. At €476 per container, the total upfront investment for container acquisition amounts to €1,352,792.

**Fig. 3:**
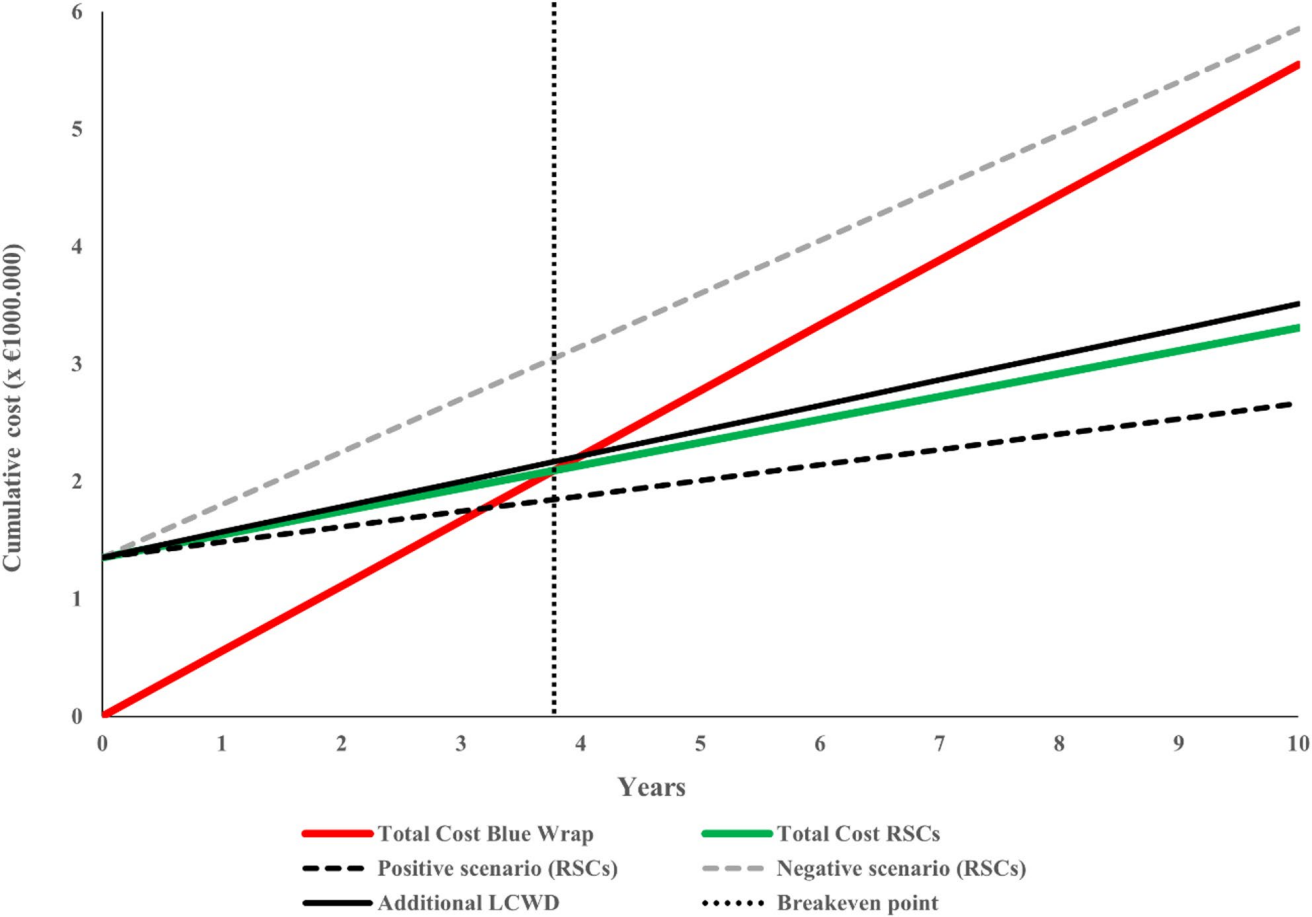
Cost comparison and breakeven analysis of blue wrap vs. RSCs

Using a cost difference of €1.78 per set in favor of RSCs, calculated from total packaging and processing costs of €0.97 per set for RSCs and €2.75 per set for blue wrap, the projected annual savings equal approximately €359,070. The €0.97 per set for RSCs excludes container acquisition costs of €0.67, as stated in Table 4, which are accounted for separately in the initial investment. As shown in Fig. 3, the upfront investment of €1,352,792 would be recovered after approximately 3 years and 9 months of use.

To assess the containers’ durability requirements, the minimum number of uses needed to achieve breakeven was calculated. The breakeven threshold was determined by dividing the unit purchase price of a container (€476) by the cost advantage per sterilization cycle (€1.78), resulting in approximately 267 cycles per container. Assuming a 10-year service life, this equates to an average of 27 cycles per container per year. In the studied CSSD, the observed mean usage rate was 71 cycles per container per year, which substantially exceeds the minimum threshold required to reach breakeven.

### Scenario analysis

The financial analysis shows that RSCs are more cost-effective than blue wrap. However, a critical question for decision-makers is how stable this result is when key assumptions are subject to variability. To evaluate the sensitivity and robustness of these results under changing parameters, the following potential variations were considered: fluctuations in the frequency of use of surgical instrument sets, adjustments in the expected lifespan of RSCs, and the acquisition of an additional LCWD (€200.000).

**Table 5:** Scenario analysis

Scenario		Cost per instrument set in €
Annual usage frequency per set	-25%	1.81
	-10%	1.66
	10%	1.53
	25%	1.56
Expected lifespan RSCs	5 years	2.52
	15 years	1.50
Additional LCWD		1.74 (+ 0.10)
Positive scenario	25% and 15 years	1.32 (-0.32)
Negative scenario	-25% and 5 years	2.90 (+ 1.36)

Table 5 presents a scenario analysis that assesses the sensitivity of RSC costs per instrument set under different conditions. This analysis includes changes in turnover rate, container lifespan, and investment in an LCWD.

The analysis shows that the cost per instrument set using RSCs varies based on annual usage frequency and container lifespan. Specifically, the lowest cost of €1.32 per instrument set was achieved with a usage frequency up to 25% above the average of 71 cycles per container per year and an extended container lifespan of up to 15 years. Conversely, decreasing usage frequency by 25%, combined with reducing the lifespan to 5 years, increased the cost to €2.90 per set. Investing in an additional LCWD added approximately €0.10 to the cost per set, raising it from €1.64 to €1.74 based on an average annual throughput of 201,725 instrument sets over a ten-year period. Across all analyzed scenarios, the cost per instrument set of using RSCs ranged from €1.32 to €2.90 per instrument set.

### Lean waste analysis

The value stream map (VSM) revealed several inefficiencies in the current sterilization workflow that resulted in non value-adding activities (muda), variability (mura), and overburden (muri). This Lean waste analysis was used not only to compare the current and future states, but also to examine the process implications of transitioning from blue wrap to RSCs. By analyzing changes across three Lean waste domains, the study identifies both the operational benefits of RSC implementation and the new challenges that may arise during implementation. Table 6 presents the observed process inefficiencies at the primary CSSD, categorized according to the three Lean waste domains. These inefficiencies served as a basis for evaluating process changes under RSC implementation in the future-state scenario.

The adoption of RSCs addressed several of the current-state inefficiencies, particularly those related to packaging waste and idle time. The elimination of blue wrap reduced the risk of damaged or contaminated packaging, thereby avoiding unnecessary re-sterilization cycles. Packaging tasks became faster and more standardized, with a 61% reduction in average time per set. Additionally, autoclave space was used more efficiently due to the standardized shape and stackability of RSCs, increasing per-cycle throughput by 25%. These changes represent a direct reduction in non-value-adding activities (muda) and demonstrate how RSC implementation alters key process steps within the CSSD workflow.

However, not all forms of waste were eliminated through RSC implementation. Variability in instrument set return timing persisted due to upstream OR scheduling practices, which resulted in inconsistent procedure end times and uneven case distributions throughout the day. This variability continued to disrupt downstream CSSD operations, causing bottlenecks in decontamination and alternating periods of idle time and workload peaks, making it challenging to sustain a steady and efficient pace across all process steps.

In terms of overburden (muri), the transition to RSCs produced mixed effects. Although the elimination of manual wrapping reduced repetitive motions and some physical strain, the weight and bulk of the containers continued to place physical demands on staff during handling and transport. Furthermore, the increased cleaning requirements associated with RSCs intensified the workload of large-capacity washer-disinfectors (LCWDs), particularly on high-volume days. This shift introduced a potential new bottleneck and source of overburden, emphasizing that efficiency gains in one part of the process may transfer workload to another if capacity constraints are not addressed.

**Table 6:** Observed inefficiencies in the CSSD workflows using Lean waste principles

Lean category	Observed issue		Future state
Non-value adding activities (muda)	Repackaging and re-sterilization	Due to damaged or contaminated blue wraps, requiring additional processing cycles.	RSCs prevent wrap damage reducing the need for re-sterilization.
	Idle time between process steps	Trays queued between washing and wrapping, and again before sterilization, leading to downtime.	Faster packaging reduces queue times between washing and wrapping; improved autoclave loading increases throughput.
Variability (mura)	Irregular return of instrument sets from hospital	Unpredictable workflows and batch arrivals from the OR caused bottlenecks and disrupted flows.	Variability remains due to upstream OR scheduling: peaks in workload still occur.
Overburden (muri)	Manual handling sets	Repetitive lifting and wrapping of heavy trays placed physical strain on staff, increasing injury risk.	Wrapping strain is eliminated but RSCs are still as heavy and require manual handling.
	Overloaded washer-disinfectors	On high-volume days, equipment operated at or beyond capacity, requiring staff to work under pressure with minimal buffer.	RSCs increase LCWD workload; risk of new bottlenecks unless mitigated by workflow design

These identified inefficiencies and waste categories provide a structured explanation for the observed differences between the two scenarios, including reductions in packaging time and improved autoclave utilization, alongside persistent variability in instrument return patterns and increased cleaning demands associated with RSC use. These observations form the basis for further interpretation of the process-level trade-offs associated with transitioning from blue wrap to reusable sterilization containers.

## Discussion

This study examined the operational and systemic implications of transitioning from single-use blue wrap to reusable sterilization containers (RSCs) in a high-throughput Central Sterile Supply Department (CSSD). By combining value stream mapping (VSM) with scenario-based capacity and cost analyses, the findings demonstrate that this transition extends beyond material substitution and has measurable effects on workflow structure, capacity utilization, and bottleneck behavior within hospital logistics systems.

### Logistical prerequisites for cost-effective RSC implementation

The operational effects discussed in this section are grounded in the VSM analysis, which illustrated how changes in packaging methods propagate through the sterilization workflow and affect process timing and equipment utilization. Previous studies have established the environmental benefits of RSCs, including reductions in packaging waste and lifecycle emissions [11, 12]. However, relatively limited attention has been paid to the operational consequences of RSC adoption within daily CSSD workflows.

This study addresses this gap by showing that RSC implementation is associated with substantial changes in process performance. Specifically, packaging time was reduced by 61%, and autoclave loading efficiency increased by 25% per cycle. These improvements directly align with Lean objectives by reducing non-value-adding activities and improving the utilization of constrained resources [18]. While minor variability in RSC packaging time may occur in practice, the standardized nature of the container-based procedure suggests that such variation is limited and unlikely to materially affect the relative cost and capacity comparisons presented in this study. Although labor time reductions do not automatically translate into immediate staff reductions, they do represent a relevant operational and economic effect in a highly standardized, high-throughput CSSD environment. In practice, released labor time can be used to absorb demand fluctuations, reduce overtime, or accommodate future volume growth without proportional increases in staffing levels. In this sense, labor efficiency gains contribute both to cost control and to system robustness. Alternatively, labor time savings may be interpreted as increased operational flexibility, enabling staff to be temporarily reallocated to other necessary tasks when required.

At the same time, the analysis revealed that RSC adoption may introduce new operational pressures. In particular, the increased demand for large-capacity washer-disinfectors (LCWDs), which are also used for cleaning transport trolleys, emerged as a potential bottleneck under peak workload conditions. Although average daily demand remained manageable, late instrument returns resulted in temporary overload of LCWD capacity. This finding highlights an operational trade-off that is largely absent from previous sustainability-focused studies but is critical for maintaining reliable high-throughput sterile processing. Prior research has similarly shown that uneven flow and infrastructure constraints are key determinants of hospital logistics performance [20].

Mitigating these risks requires targeted, system-level interventions. Potential strategies include increasing LCWD capacity, adjusting instrument return schedules, or implementing staggered collection rounds in coordination with operating room teams. This reinforces earlier findings that Lean improvements in healthcare are most effective when scheduling and workflow decisions are coordinated across departments rather than optimized locally [21]. Studies by Göras et al. (2020) and Ribes-Iborra et al. (2022) likewise emphasize the interdependence of perioperative domains and the importance of cross-departmental alignment to sustain reliable process flows [22, 23]. Together, these insights support a system-wide approach in which CSSD operations, surgical scheduling, and logistics services are jointly managed.

From a theoretical perspective, this study contributes to the literature by explicitly linking Lean thinking to sustainability transitions through operational and capacity-based modeling. Whereas many studies treat Lean implementation and environmental sustainability as parallel but separate initiatives, the present findings demonstrate their mutual dependence in complex clinical logistics environments. By quantifying the operational implications of equipment investments, usage intensity, and realistic asset lifespans, this work provides actionable insights for both researchers and hospital practitioners considering container-based sterilization systems.

### Managerial implications

The managerial implications of these findings are substantial. Prior analyses of RSC adoption have largely focused on material, environmental, or unit-cost outcomes [10–12], often underestimating the extent of operational adaptation required for successful implementation. This study demonstrates that gains in packaging efficiency and autoclave utilization depend on whether newly introduced capacity constraints in washer-disinfector processes are proactively addressed.

The financial analysis supports the economic viability of RSCs under realistic operating conditions. The per-set sterilization cost decreased from €2.75 to €1.64, with the investment reaching breakeven within four years under baseline assumptions. Even when additional infrastructure investments were included, RSCs remained cost-effective over their operational lifespan. These findings align with logistics and operations research emphasizing that sustainable investments should be evaluated within the context of demand stability, capacity buffers, and system resilience [24, 25]. The scenario analyses further illustrate how investment size, utilization patterns, and equipment lifespan jointly influence the breakeven point, making the results directly relevant for hospital management decision-making.

From a policy perspective, the results indicate that sustainability and operational efficiency can be mutually reinforced, provided that supporting systems are aligned with altered work patterns. This is particularly relevant considering the Dutch Green Deal for Sustainable Healthcare and broader European climate targets [26]. CSSDs play a critical role in achieving these objectives, but only if they are recognized as integral components of hospital logistics systems rather than isolated technical units. The benchmarks presented in this study offer decision-makers a structured basis for evaluating sustainable sterilization strategies.

### Limitations and future research

Several limitations should be considered when interpreting these findings. First, the analysis focused on CSSD-level operations and did not systematically assess downstream effects on operating room workflows, surgical waiting times, or patient outcomes. Second, generalizability may be limited for smaller or less standardized organizations, where infrastructural thresholds and timelines for realizing efficiency gains may differ.

The analysis further assumed stable demand patterns and consistent equipment availability. Although this assumption is supported by the use of cyclic operating room schedules in many hospitals, factors such as emergency cases, staff shortages, and technical failures were not explicitly modeled and may influence real-world performance. While staff input informed the value stream mapping, the study did not include a structured qualitative assessment of staff experience, ergonomic impact, or implementation barriers. Future research could address these aspects through interviews, ethnographic observation, or survey-based approaches.

Finally, although the use of three randomly selected, non-consecutive observation weeks enhanced representativeness compared with continuous short-term sampling, rare extreme demand peaks and long-term disruptions were not fully captured. Future studies could incorporate multi-year operational datasets or discrete-event simulation models to dynamically evaluate capacity requirements, buffer sizing, and bottleneck behavior under variable demand conditions, thereby complementing the static, scenario-based approach used here.

## Conclusion and recommendations

This study demonstrates that transitioning from single-use blue wrap to reusable sterilization containers (RSCs) in a high-throughput CSSD generates measurable environmental, financial, and operational benefits. By integrating Lean principles with capacity and cost modeling, we show that sustainable material innovations must be implemented within a systemic framework, in which emerging bottlenecks are promptly identified, and coordination between clinical, logistical, and support teams is actively maintained.

The adoption of RSCs substantially reduces packaging waste, packaging time (by 61%), and cost per sterilization cycle, while increasing autoclave loading efficiency. However, it also introduces new operational challenges, particularly regarding the increased demand for large-capacity washer-disinfectors. Our findings highlight that the full advantages of RSC implementation are realized only when hospitals adopt a holistic strategy that addresses infrastructure requirements, workflow coordination, and interdepartmental collaboration.

The practical benchmarks and scenario-based analyses provided in this study can guide hospital leadership, logistics teams, and policymakers in making informed, sustainable, and future-proof investment decisions. These insights illustrate that sustainability and efficiency are not inherently in conflict; on the contrary, when implemented thoughtfully, environmentally responsible innovations can reinforce operational performance.

Future research should extend these findings by examining the broader implications of RSC implementation on clinical practice, staff workload and ergonomics, and patient outcomes, thereby capturing the impact of such interventions across the entire care delivery process.

## Supplementary Information

Below is the link to the electronic supplementary material.

Supplementary Material 1

## Data Availability

The data underlying this study are not publicly available due to the inclusion of proprietary and commercially sensitive company information, but are available from the corresponding author on reasonable request.

## References

[1] OECD/EU. Health at a glance: Europe 2022 – State of health in the EU cycle. Paris: OECD Publishing. 2022. Available from: 10.1787/507433b0-en.

[2] Varkevisser G, Maarse H, Jeurissen P. Sustainability and resilience in the dutch health system: a PHSSR Netherlands country report. Rotterdam: Erasmus University Rotterdam; 2023. Available from: https://pure.eur.nl/files/91080609/WEF_PHSSR_Netherlands_Report_2023.pdf.

[3] Steenmeijer RIVM, Pieters MA, Houtman LI. MM. Het effect van de zorgsector op het milieu. Bilthoven: Rijksinstituut voor Volksgezondheid en Milieu (RIVM); 2023.

[4] Kagoma Y, Stall N, Rubinstein E, Naudie D. People, planet and profits: the case for greening operating rooms. CMAJ. 2012;184(17):1905–11. 22664760 10.1503/cmaj.112139 PMC3503903

[5] MacNeill AJ, Lillywhite R, Brown CJ. The impact of surgery on global climate: a carbon footprinting study of operating theatres in three health systems. Lancet Planet Health. 2017;1(9):e381–6. 29851650 10.1016/S2542-5196(17)30162-6

[6] Pan W, Hu J, Yi L. Mental state of central sterile supply department staff during COVID-19 epidemic and CART analysis. BMC Health Serv Res. 2020;20(1):1–7. 10.1186/s12913-020-05864-5 PMC7609829 33148244

[7] Chang CD, Brenner MJ, Shuman EK, Kokoska MS. Reprocessing standards for medical devices and equipment in otolaryngology. Otolaryngol Clin North Am. 2019;52(1):173–83. 30262168 10.1016/j.otc.2018.08.014

[8] McGain F, Jarosz KM, Nguyen MNHH, Bates S, O’Shea CJ. Auditing operating room recycling. Case Rep. 2015;5(3):47–50. 10.1213/XAA.0000000000000097 26230308

[9] Azouz S, Boyll P, Swanson M, Castel N, Maffi T, Rebecca AM. Managing barriers to recycling in the operating room. Am J Surg. 2018;217(4):634–8. 29958657 10.1016/j.amjsurg.2018.06.020

[10] Krohn M, Fengler J, Mickley T, Flessa S. Analysis of processes and costs of alternative packaging options of sterile goods in hospitals – a case study in two German hospitals. Health Econ Rev. 2019;9(1):1–8. 30656503 10.1186/s13561-018-0218-2 PMC6734388

[11] Rizan C, Bhutta MF, Reed M, Lillywhite R. The carbon footprint of reprocessing surgical instruments in the National Health Service in England: a carbon footprint analysis. J Hosp Infect. 2022;111:108–16.

[12] Friedericy HJ, van Egmond CW, Vogtländer JG, van der Eijk AC, Jansen FW. Reducing the environmental impact of sterilization packaging for surgical instruments in the operating room: a comparative life cycle assessment of disposable versus reusable systems. Sustainability. 2021;14(1):430.

[13] Agnetis A, Coppi A, Corsini M, Dellino G, Meloni C, Pranzo M. Long term evaluation of operating theater planning policies. Oper Res health care. 2012;1:95–104. 10.1016/j.orhc.2012.10.001.

[14] Rahimi I, Gandomi A. A Comprehensive Review and Analysis of Operating Room and Surgery Scheduling. Arch Comput Methods Eng. 2020;28:1667–88. 10.1007/s11831-020-09432-2.

[15] Cardoen B, Demeulemeester E, Beliën J. Operating room planning and scheduling: a literature review. Eur J Oper Res. 2010;201:921–32. 10.1016/j.ejor.2009.04.011.

[16] Ohno T. Toyota Production System: Beyond Large-Scale Production. Portland: Productivity; 1988.

[17] Womack JP, Jones DT. Lean Thinking: Banish Waste and Create Wealth in Your Corporation. 2nd ed. New York: Free; 2003.

[18] Crane J, Noon C. Value stream mapping. The Definitive Guide to Emergency Department Operational Improvement. Boca Raton: CRC; 2017.

[19] Lasa I, Laburu C, de Castro Vila R. An evaluation of the value stream mapping tool. Bus Process Manag J. 2008;14(1):39–52.

[20] Fallahnezhad M, Langarizadeh M, Vahabzadeh A. Key performance indicators of hospital supply chain: a systematic review. BMC Health Serv Res. 2024;24:1610. 10.1186/s12913-024-11954-5. 39696247 10.1186/s12913-024-11954-5 PMC11654143

[21] Tortorella G, Fogliatto F, Anzanello M, Marodin G, Garcia M, Esteves R. Making the value flow: application of value stream mapping in a Brazilian public healthcare organisation. Total Qual Manag Bus Excell. 2017;28(13–14):1544–58.

[22] Göras C, Nilsson U, Ekstedt M, et al. Managing complexity in the operating room: a group interview study. BMC Health Serv Res. 2020;20:440. 10.1186/s12913-020-05192-8. 32430074 10.1186/s12913-020-05192-8 PMC7236109

[23] Ribes-Iborra J, Segarra B, Cortés-Tronch V, et al. Improving perioperative management of surgical sets for trauma surgeries: the 4S approach. BMC Health Serv Res. 2022;22:1298. 10.1186/s12913-022-08671-2. 36307812 10.1186/s12913-022-08671-2 PMC9615625

[24] Uthayakumar R, Priyan S. Pharmaceutical supply chain and inventory management strategies: optimization for a pharmaceutical company and a hospital. Oper Res Health Care. 2013;2(3):52–64.

[25] Biagi M, Carnevali L, Santoni F, Vicario E. Hospital inventory management through Markov decision processes. In: Cavalieri S, Reed R, editors. Applications of simulation and modeling. Cham: Springer; 2018. pp. 87–103.

[26] Green Deal Duurzame Zorg. Green Deal duurzame zorg. 2022. Available from: https://www.greendealduurzamezorg.nl/.

