# Supplementary material for: Evaluating the implementation of rigid sterilization containers in a Dutch central sterile supply department: a scenario-based cost and workflow analysis
Source: BMC Health Serv Res. 2026 Mar 26;26:633. doi: 10.1186/s12913-026-14391-8 (PMC13141316; doi:10.1186/s12913-026-14391-8)
Supplement: Supplementary file 1 — Supplementary Material 1 [file 12913_2026_14391_MOESM1_ESM.docx]

**Appendix A - Topics discussed during on-site observations**

This appendix provides an overview of the topics addressed during informal consultations with staff at both CSSD sites. These conversations were conducted during on-site observations and focused exclusively on operational workflows and technical routines. No personal or medical data were collected.

| **Topic Area** | **Description** |
| --- | --- |
| Packaging workflow | Steps involved in wrapping or containerizing instrument sets |
| Time allocation | Average time spent on packaging tasks per set |
| Equipment usage | Utilization of washer-disinfectors and autoclaves |
| Staff task distribution | Roles and responsibilities during the sterilization processes |
| Bottlenecks and inefficiencies | Observed delays, redundancies or workload imbalances |
| RSC implementation feedback | Staff experiences and practical insights regarding the use of blue wrap and reusable sterilization containers |
| Validation of modeled assumptions | Confirmation of time estimates, and workflow parameters used in analysis |

|  |  |
| --- | --- |
|  |  |

*Note: These consultations were informal and context-specific, aimed at clarifying observed practices and validating operational data.*
